# Diet–vaccine interactions: SQM Iron and *Salmonella* vaccination shape poultry gut microbiota

**DOI:** 10.1128/aem.00127-26

**Published:** 2026-04-14

**Authors:** Eldon O. Ager, Colette A. Nickodem, Jessica Brown, Joshua Jendza, Eric Neeno-Eckwall, Marisa Schuldes, Dana K. Dittoe, Jessica L. Hite

**Affiliations:** 1 Department of Integrative Biology, University of Wisconsin-Madison 5228, Madison, Wisconsin, USA; 2 Department of Pathobiological Sciences, University of Wisconsin-Madison 5228, Madison, Wisconsin, USA; 3 Department of Animal and Dairy Sciences, University of Wisconsin-Madison 5228, Madison, Wisconsin, USA; 4 QualiTech, LLC 734810 , Chaska, Minnesota, USA; 5 Department of Animal Science, University of Wyoming 4416, Laramie, Wyoming, USA; Universita degli Studi di Napoli Federico II, Portici, Italy

**Keywords:** SQM Iron, nutritional management, poultry, *Salmonella*, AviPro Megan Vac 1

## Abstract

Vaccines to prevent *Salmonella* are rapidly gaining traction in the poultry industry. Yet how these interventions interact with other management strategies to influence the broader gut microbial community, rather than *Salmonella per se,* remains poorly understood. Understanding these effects is critical because shifts in the microbiome can alter nutrient metabolism, immune function, and pathogen dynamics in ways that could either enhance or suppress management strategies aimed at improving bird health and food safety. Here, we examine how a live-attenuated *Salmonella* vaccine (AviPro Megan Vac 1) and iron supplementation, two widely adapted management strategies, individually and jointly shape the cecal microbiome of broiler chickens. Specifically, we compare standard iron supplements (FeSO_4_) with a polysaccharide-complexed iron supplement (SQM Iron) which uses a complexation process that “hides” iron from *Salmonella* via time- and tissue-specific release of this critical nutrient. Using 16S rRNA gene sequencing, we found that while overall microbial diversity was unchanged, both interventions induced reproducible shifts in community composition, including enrichment of taxa linked to fermentation and short-chain fatty acid production. Vaccination alone promoted *Bacillota* genera associated with gut homeostasis, whereas iron supplementation altered competitive dynamics by reducing *Streptococcus* and favoring several rare taxa. Strikingly, combining these treatments suppressed several beneficial fermentative genera, and *Staphylococcus* was markedly increased, revealing nonadditive effects. These findings illuminate the potential of integrated strategies, combining immune stimulation with precision micronutrient supplementation, to improve poultry health and food safety. However, these results also underscore the intricate microbial trade-offs that must be carefully navigated to avoid unintended consequences in modern production systems.

**IMPORTANCE:**

Globally, non-typhoidal *Salmonella* (NTS) is a persistent food safety challenge and pre-harvest control is an industry priority. While *Salmonella* vaccines are rapidly gaining adoption, their interactions with other common management practices such as nutritional strategies remain unclear. Iron metabolism is particularly important, as it influences host immunity, pathogen colonization, and shapes the gut microbiome. This study investigates how live-attenuated *Salmonella* vaccine (AviPro Megan Vac 1) and iron-based nutritional management interact to shape the cecal microbiota of broiler chickens. Specifically, we focus on SQM Iron, with a complexation process that enables time- and tissue-specific release of this critical nutrient. Our findings indicate that targeted combinations of immune stimulation and micronutrient supplementation can selectively remodel the poultry gut microbiome, with potential implications for nutrient utilization, microbial metabolism, and integrated, non-antibiotic approaches to reduce *Salmonella* burden while supporting flock health.

## INTRODUCTION

Non-typhoidal *Salmonella* (NTS) continues to impose significant public health and economic burdens in the United States, causing an estimated 1.35 million infections, 26,000 hospitalizations, and 400 deaths annually (1). Many of these infections are linked with poultry (2). Despite longstanding investments in sanitation, vaccination, and antimicrobial interventions, these strategies have not sustainably reduced *Salmonella* prevalence in poultry or foodborne illnesses in humans (3, 4). As global demand for affordable poultry products increases, especially in major export markets like the United States, the need for integrative, non-pharmacological control strategies is increasingly urgent (5).

Nutritional management represents a promising approach that could work alongside vaccination to improve both poultry health and food safety outcomes (6, 7). Among nutritional factors, iron metabolism is particularly compelling given its essential role for both hosts and pathogens. Iron plays an integral role in numerous physiological processes such as respiration, gene regulation, and DNA biosynthesis (8, 9). Iron is also crucial for intestinal integrity, beneficial gut microbes, and immune functions (10, 11). Consequently, hosts have evolved numerous mechanisms to regulate the distribution, accumulation, and bioavailability of this essential nutrient. For instance, hosts sequester iron as a potent defense against pathogens, in a process termed nutritional immunity (12–15). This hypoferremic response is a critical component of innate immunity, sequestering iron within host cells and tissues to restrict its availability to invading pathogens. The importance of this sequestration is underscored by the fact that individuals with iron overload or deficiency, resulting from either mutations in iron homeostasis or malnutrition, exhibit a significantly heightened susceptibility to infection (16, 17).

Iron is particularly critical for pathogen virulence and tissue colonization (6, 8, 18). For instance, iron uptake and efflux products released by *S. enterica* into the host environment are essential for evading host immunity, tissue adhesion, and successful pathogenesis. Because iron is critical for numerous bacterial pathogens, including *Salmonella*, it can be an effective therapeutic target to limit pathogen colonization and invasion of the gut (19). However, any iron-based nutritional intervention must achieve a balance between starving the pathogen and maintaining host health. Prolonged iron sequestration can, for example, cause anemia as well as poor growth and production in poultry (18, 20). Several approaches to reduce the negative by-products of iron sequestration through nutritional supplements have been tested, including attempts to alleviate inflammation and anemia with iron supplementation. Yet many of these treatments present additional challenges, often causing rapid growth of gut pathogens and oxidative stress (21, 22).

**Fig 1:**
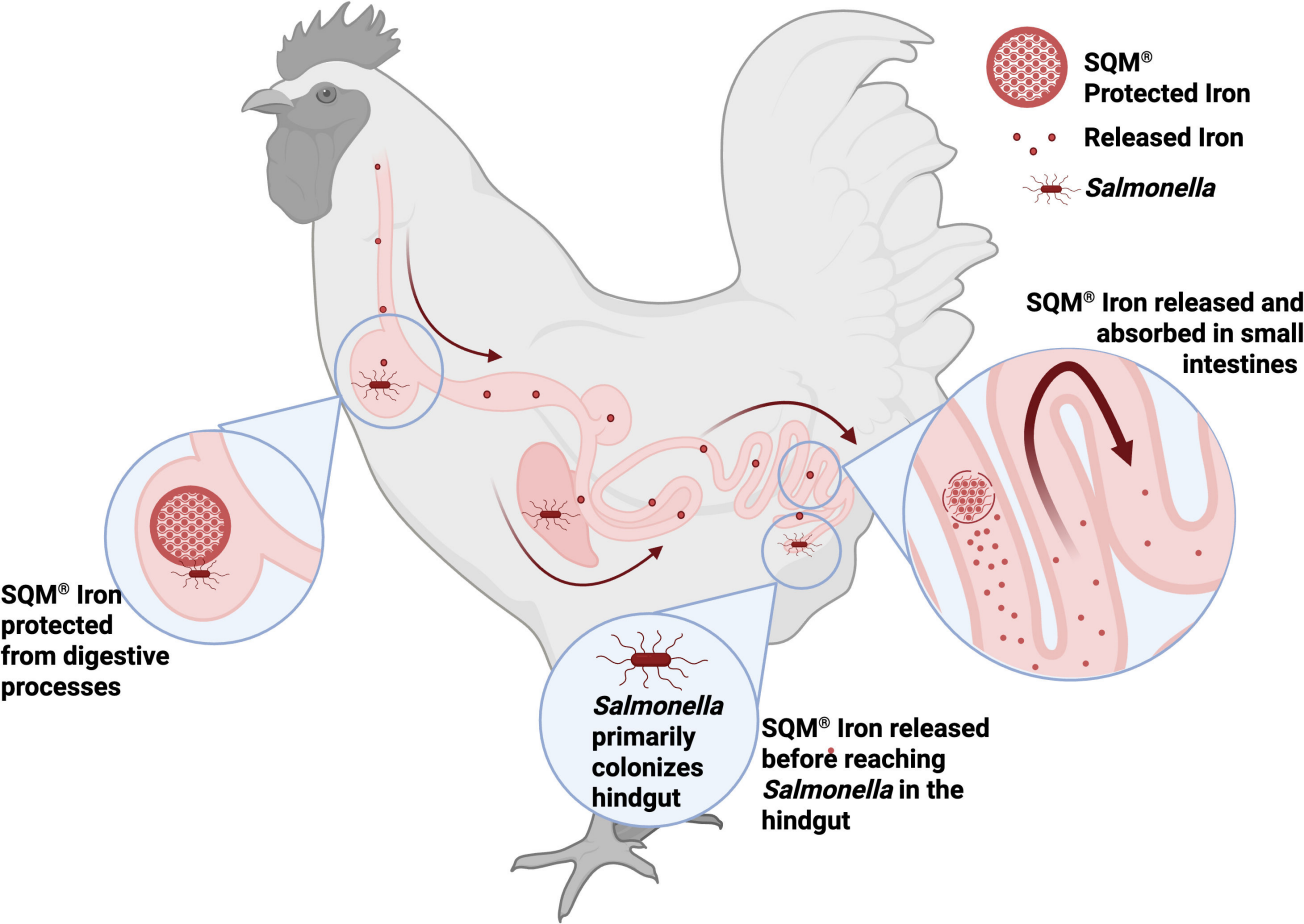
Overview of SQM Iron. Time-release encapsulation technologies improve the bioavailability of iron supplements by ensuring that they remain in a non-absorbable form until they reach the physiological site of maximum absorption. Complexation of organic trace minerals (such as iron) to organic ligands (such as polysaccharide biopolymers) improves bioavailability to the animal. How? By stabilizing the charge state of the mineral, thereby reducing the likelihood of the mineral engaging in undesirable reactions that would render it insoluble and unavailable to the host. Minerals already complexed to soluble/degradable ligands do not interact with phytic acid in the stomach where phytic acid precipitation reactions are most likely to occur and can later be released from the ligand at the site of absorption by endogenous and exogenous carbohydrases, which are abundant in the small intestine. This protection of trace minerals increases the proportion of ingested iron that is absorbed by the host, not the pathogen. (Created by D. Dittoe with BioRender.com. [https://BioRender.com/t5n4y0x])

To overcome these challenges, time-release encapsulation technologies, such as SQM Iron (QualiTech, MN USA), offer a targeted solution by balancing host iron requirements with pathogen control. SQM Iron is an FDA-approved polysaccharide-iron complex that uses PolyTransport technology to chelate iron ions with polysaccharide ligands. Because more than one biopolymer chain in SQM Iron can bind to a single iron atom, this compound is classified as a complex under FDA rules. This technological innovation ensures controlled, site-specific release within the gastrointestinal tract (20, 23). This time-release mechanism stabilizes iron during digestion while preventing excess iron in the upper gut. These changes improve the bioavailability of iron in the small intestine, the primary site of iron absorption in monogastric animals like poultry. By maximizing absorption where it is most needed and minimizing unabsorbed iron reaching the ceca, a key site for *Salmonella* colonization, SQM Iron could address both nutritional and food safety goals (Fig. 1). By improving nutrient utilization while minimizing iron availability to pathogens, it holds particular promise for simultaneously optimizing bird health and limiting *Salmonella* proliferation.

However, despite their promise, these nutrtional intervetion strategies have yet to be widely adopted, partly because their interactions with other management practices, especially vaccination and microbiome dynamics, remain poorly understood (24–26). Here, we take a first step toward evaluating these dynamics by examining the combined effects of vaccination and SQM Iron supplementation on the cecal microbiome. Specifically, we focus on interactions with a live-attenuated *Salmonella* vaccine, AviPro Megan Vac 1. This vaccine is administered to enhance the immune response against and reduce colonization of *Salmonella* serovars Typhimurium, Enteritidis, and Heidelberg within the chicken digestive tract and internal organs (27). We hypothesized that unlike inactivated vaccines, live-attenuated vaccines could *potentially* have a direct effect on the microbiome, especially in response to dietary treatments.

To test this hypothesis, we combined experiments with female broiler chickens, 16S sequencing, and bioinformatics to address two key questions. (i) Do live-attenuated vaccines, that only transiently colonize the intestinal tract, perturb microbiome composition in ways that persist even after those strains have dissipated? (ii) Do resource-driven changes in the gut microbiome mirror those caused by transient vaccine strain colonization, and how do these processes interact? By evaluating the individual and combined effects of vaccination and nutritional iron modulation, our goal is to identify synergistic or antagonistic effects relevant to integrated strategies to control *Salmonella*. Our results provide foundational insight into how vaccines and nutritional management interact to shape microbial communities, especially rare taxa (28, 29).

## MATERIALS AND METHODS

### Experimental design

**Fig 2:**
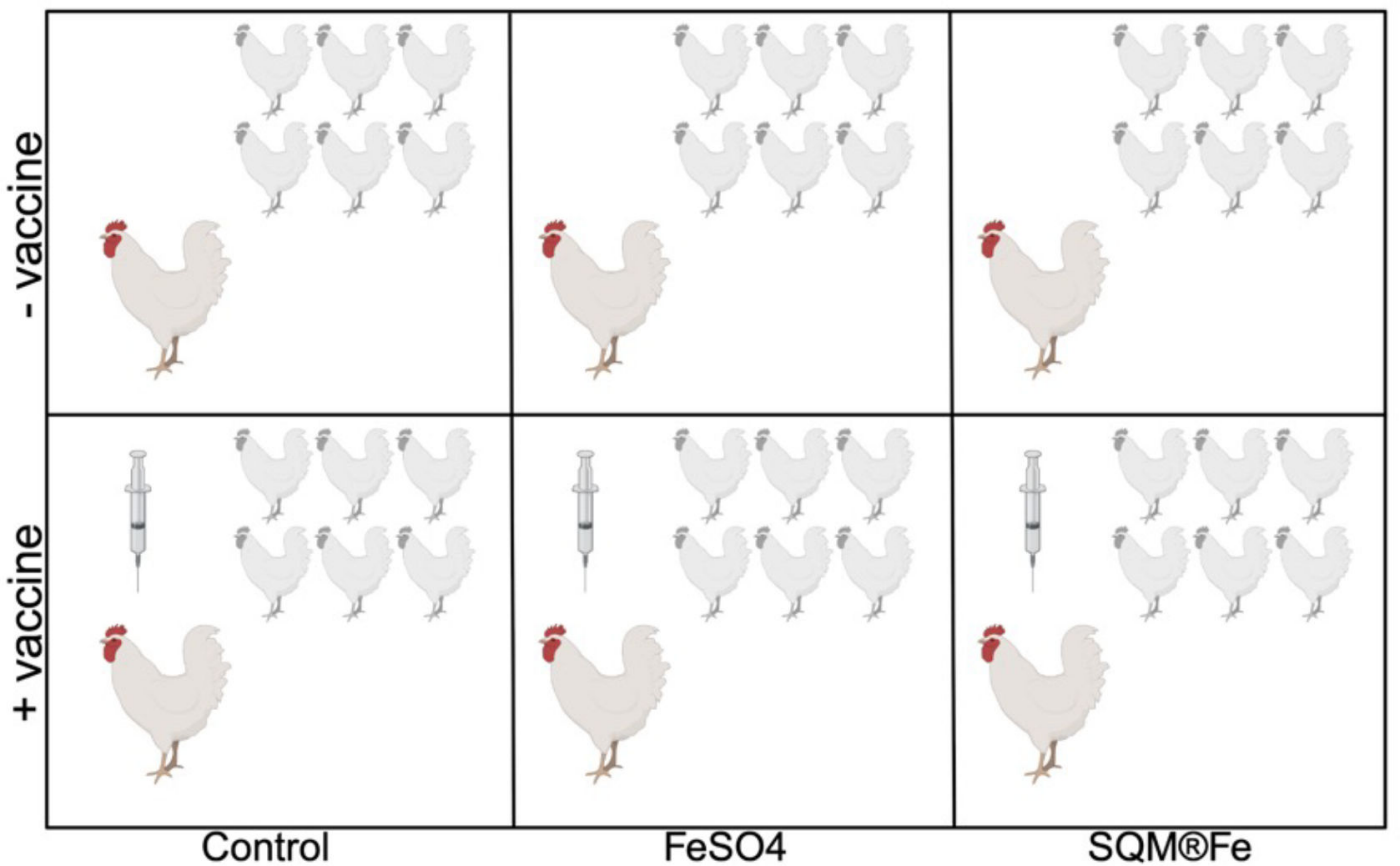
Overview of experimental design. Ross 708 female broilers were allocated across six treatment groups in a fully crossed 2 × 3 factorial design (vaccination status: vaccinated vs. unvaccinated; supplementation: control, FeSO₄, SQM Iron). Birds were housed in 5 replicate pens per treatment group with each pen containing 7 birds (35 birds per treatment group, 210 birds total). Birds received feed and water *ad libitum* throughout the study. Cecal samples were collected on day 49. Due to logistical constraints, we sampled one bird per pen (hence each treatment had 5 replicates per treatment, *n* = 5 birds per each treatment). The figure below highlights the seven birds in each pen, with one focal bird per pen selected for sampling.

To investigate the interaction between iron supplementation and vaccination on the cecal microbiome of chickens, we conducted a 49-day feeding trial using day-old Ross 708 female broilers (*N* = 210) in a 2 × 3 factorial design (Fig. 2). Treatments included two vaccination groups (vaccinated with AviPro Megan Vac 1 or non-vaccinated) and three iron sources (control without supplemental iron, FeSO₄, or SQM Iron from QualiTech, Inc.), with all iron sources included at 60 ppm. Vaccinated birds received AviPro Megan Vac 1 via spray application (1.0 mL per 100 chicks) on day 0, with a booster administered via drinking water on day 14, per manufacturer guidelines. To prevent cross-contamination, vaccinated and non-vaccinated birds were housed in separate sections of the facility, maintaining a minimum distance of 20 feet.

Upon arrival at the Virginia Diversified Research facility (VDRC), Harrisonburg, VA, USA, birds were individually weighed and randomly assigned to treatment groups using a randomized complete block design, with five replicate pens per treatment and seven birds per pen. Birds were housed in 1.5 × 1.5 m floor pens bedded with fresh wood shavings (0.074 m² per bird) under standard lighting conditions, with *ad libitum* access to water and mash-form feed provided through a three-phase feeding program: starter (days 0–14), grower (days 14–28), and finisher (days 28–49). The feed was formulated by Hooge Consulting Service, LLC, (Eagle Mountain, UT) according to industry standards and guidelines established by the National Research Council to meet or exceed nutrient requirements for chickens (30). Each dietary phase was formulated from a common basal diet with iron source adjustments according to treatment (Table S1). At day 49, one bird per pen was selected for cecal sampling to assess the effects of vaccination and iron supplementation on gut microbial composition through metagenomic analysis.

The animal care and use procedures adhered to the Guide for the Care and Use of Agricultural Animals in Research and Teaching and were approved by the University of Wisconsin-Madison Institutional Animal Care and Use Committee (IACUC protocol #B00000944-AM008). All experimental procedures, including vaccination, dietary supplementation, and sample collection, were designed to minimize animal discomfort and distress and followed established standards for poultry care and husbandry. Birds were monitored daily for general health and welfare, with veterinary oversight throughout. Housing conditions, feeding regimens, and handling protocols complied with institutional animal care policies and federal regulations to ensure the humane treatment of animals throughout the study.

### DNA extraction and quality assessment

Total genomic DNA was extracted from cecal content samples to enable downstream 16S rRNA gene amplification and sequencing. The DNA from cecal samples (0.5 g) was isolated and purified using the QIAamp PowerSoil Pro kit (Qiagen catalog #47,016) following the manufacturer’s protocol exactly, with final elution performed in 100 μL of the elution buffer. DNA concentration and purity were assessed using a NanoDrop spectrophotometer (model ND-1000), with acceptable samples exhibiting 260/280 ratios between 1.8 and 2.0 and 260/230 ratios above 1.5. Following quality assessment, all DNA samples were normalized to 10 ng/μL using the kit elution buffer to ensure consistent template concentrations for subsequent PCR amplification.

### 16S rRNA amplicon sequencing

High-throughput sequencing of the 16S rRNA gene was performed to characterize the cecal microbiome composition. The V4 hypervariable region was amplified using dual-indexed primers described previously (31). PCRs were performed in 25 μL volumes containing 19 μL AccuPrime Pfx SuperMix (Invitrogen catalog #12,344,040), 1 μL each of 10 μM forward and reverse primers, 2 μL template DNA (10 ng/μL), and 2 μL DMSO. Thermocycling conditions included initial denaturation at 95°C for 5 min, followed by 35 cycles of 95°C for 30 s, 55°C for 30 s, and 72°C for 60 s, with final extension at 72°C for 5 min. PCR amplification was conducted using 35 cycles instead of 30 as described in the protocol for the dual-indexing approach (31). While recent studies suggest that cycle numbers in the range from 25 to 35 do not significantly affect richness or beta-diversity patterns (32), we acknowledge that this amplification represents a potential source of technical variation affecting relative abundance estimates. PCR products were verified by 1.5% agarose gel electrophoresis and then normalized using SequalPrep Normalization Plates (Invitrogen A1051001) with 20 μL elution buffer. Normalized amplicons were pooled and quantified by qPCR using the KAPA Library Quantification Kit (Roche KAPA code: KK4824) and Qubit 1x dsDNA HS Assay Kit (Illumina). The final library was diluted to 6 pM containing 10% PhiX control (Illumina FC-110-3001) and sequenced on an Illumina MiSeq platform using V2 chemistry (Illumina MS-102-2003) to generate paired-end 250 bp reads.

### Sequencing output, preprocessing, and taxonomic assignment

Quality filtering and denoising of demultiplexed sequencing data yielded a total of 617,853 paired-end reads with a median of 13,846 (IQR: 9,137–26,942) per sample. The FASTQ files were processed using the DADA2 plugin within QIIME2 (v2024.2.0) via the denoise-paired command, which performs quality filtering, denoising, merging of paired-end reads, and chimera removal in a single step (33). Specific parameters used for this step included trimming the first 8 bases from forward reads and 29 bases from reverse reads, and truncating forward and reverse reads at 232 and 156 bases, respectively. Representative sequences were taxonomically classified using QIIME2’s feature-classifier classify-sklearn command with a pretrained naive Bayes classifier trained against the Silva 138 99% clustered reference sequence database, using a confidence threshold of 0.7 (34–36). This initial data set underwent further processing to ensure data quality, including the removal of reads classified as mitochondrial or chloroplast sequences and exclusion of low-abundance amplicon sequence variants (ASVs) that appeared in fewer than 0.001% of samples (34–37).

After these quality control steps, 90,086 high-quality reads remained, classified into 893 unique ASVs across 30 samples. To standardize sequencing depth for diversity comparisons, we used rarefaction to 2,906 sequences per sample. This approach retained the maximum number of samples and excluded two technical replicates with insufficient counts. Rarefaction curves plateaued at this sequencing depth (Fig. S1), confirming sufficient coverage for detecting bacterial diversity across treatment groups. The most abundant sequence from each ASV was used as the representative sequence for further taxonomic classification and analysis. On average, samples contained 195 ASVs post-rarefaction.

All downstream statistical analyses and visualizations were performed in R using phyloseq, ggplot2, and vegan packages (Mac version 4.4.2) (38–40). One sample (CN223 a Vaccinated/Iron Control sample) was excluded as a quality-control outlier prior to final analyses. CN223’s rarefied community was unusually dominated by Bifidobacterium (~29.9% of rarefied reads), a pattern not observed in the other Vaccinated + Control birds (near 0%), and this atypical dominance was reflected in both markedly reduced alpha diversity (Shannon = 3.999; observed ASVs = 54) and strong beta-diversity separation (largest within-group dissimilarity under both Bray–Curtis and Jaccard). The cleaned metadata were then synced with the feature table, yielding 29 samples for downstream analyses.

### Alpha diversity

To assess within-sample microbial diversity, we calculated two metrics: the number of observed ASVs (richness) and Shannon diversity using QIIME 2 (v2024.5) (41). To evaluate the individual and interactive effects of vaccination and iron supplementation on Shannon diversity (42–44), we applied the Scheirer–Ray–Hare test, an extension of Kruskal-Wallis for a two-dimensional non-parametric analog of the two-way ANOVA (45). This approach was selected due to the non-normal distribution of Shannon values, making parametric alternatives inappropriate.

### Beta diversity

We quantified between-sample diversity using Bray–Curtis dissimilarity (46), calculated in QIIME 2 (v2024.5) (41). We applied non-metric multidimensional scaling (NMDS) based on both Bray–Curtis dissimilarity (relative abundance) and Jaccard β-diversity (presence/absence) to visualize differences in bacterial community composition across samples. NMDS was performed using the metaMDS() function in the *vegan* package (version 2.8-0) (40, 43, 47), providing an ordination of community composition without assumptions about underlying taxonomic relationships. Dimensionality optimization was conducted by comparing stress values across 1–6 dimensions, selecting a 4D NMDS solution (stress = 0.095) for its balance between interpretability and model fit (Fig. S2). For Bray–Curtis, stress decreased from 0.344 (1D) to 0.095 (4D), and for Jaccard, from 0.283 (1D) to 0.081 (4D). A four-dimensional solution was selected for both metrics based on stress values below 0.10, substantial improvement over lower-dimensional models, and consistent algorithm convergence.

To examine the effects of vaccination, iron supplementation, and their interaction on beta diversity, we applied permutational multivariate analysis of variance (PERMANOVA) on both Bray–Curtis and Jaccard β-diversity matrices using the adonis2 function from the *vegan* package with 999 permutations (40). PERMANOVA models estimated Pseudo-*F* values and *P*-values for each main effect and interaction term (48–51). Pairwise differences between treatment groups were further evaluated using pairwise.adonis() with Bonferroni correction for multiple comparisons (52). These statistical results complemented visual patterns observed in NMDS ordinations and confidence ellipses, supporting interpretation of treatment effects on microbial community structure.

Finally, to examine specific taxonomic differences in microbial composition across treatment groups, we employed ANCOM-BC2 (Analysis of Compositions of Microbiomes with Bias Correction 2) (53) using the ANCOMBC package (version 2.8.1). This general framework for multigroup differential abundance analysis incorporates covariate adjustments and interaction effects. ANCOM-BC2 also accounts for both sample-specific and taxon-specific biases, the latter of which is particularly important due to variable sequencing efficiencies that can lead to preferential detection of certain taxa. This bias correction improves control of the false discovery rate (FDR) and enhances the interpretability of log-fold change estimates (53). Here, genus-level taxonomic abundance data were exported from QIIME2 (level-6) and subjected to quality control filtering. Genera were retained only if they had valid NCBI taxonomic classifications, and taxonomic names were updated using the NCBI Taxonomy Database to reflect current scientific nomenclature. Samples with fewer than 1,000 total sequence counts were excluded to ensure adequate sequencing depth. Genera present in fewer than 10% of samples were removed to focus the analysis on the most prevalent taxa. After filtering, the final data set included 84 validated genera across 29 samples.

### Differential abundance analysis

Differential abundance testing was conducted using ANCOM-BC2 that tested for the main effects of vaccination status (vaccinated vs unvaccinated), iron supplementation (control, FeSO₄, SQM Iron), and their interaction. Statistical significance was determined using the Benjamini-Hochberg procedure to correct for multiple testing, with a false discovery rate threshold of *q* < 0.05. Effect sizes were interpreted based on log₂ fold change thresholds, with values less than 0.5 considered small, values between 0.5 and 1.0 considered medium, and values ≥ 1.0 considered large, consistent with commonly accepted standards for biological relevance. To enhance statistical robustness, the analysis incorporated prevalence filtering (≥10%), library size filtering (≥1,000 counts), and structural zero detection. In total, 29 samples were analyzed, comprising 84 genera after filtering. The factorial design included three iron treatment groups and two vaccination statuses, resulting in 252 individual coefficient tests (84 genera × 3 effects).

## RESULTS

### Alpha diversity

**Fig 3:**
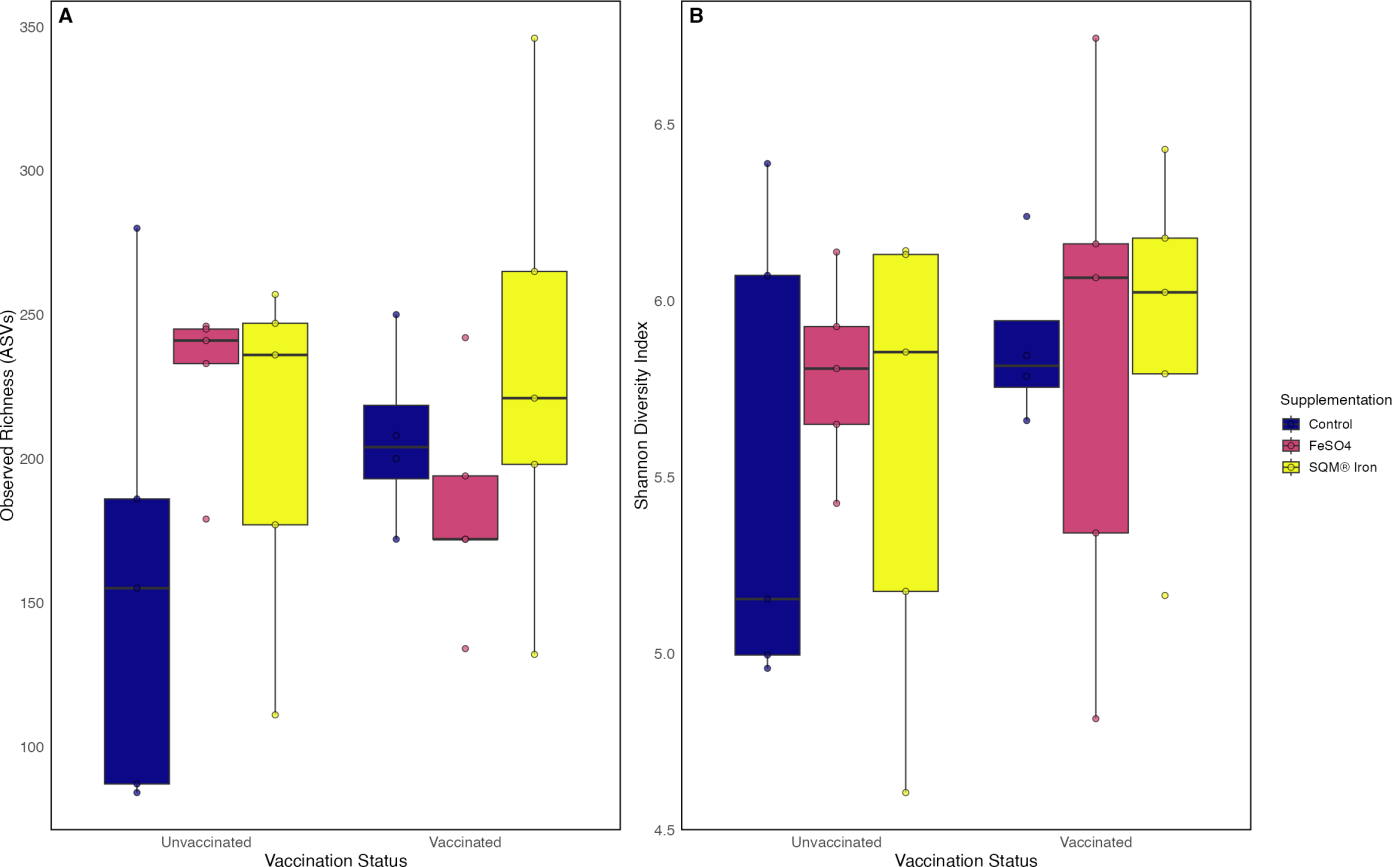
Vaccination and iron supplementation carry minor effects on the alpha diversity of the cecal microbiome of female broilers. (**A**) Observed richness (amplicon sequence variants, ASVs) and (**B**) Shannon diversity index. Neither vaccination, iron supplementation, or their interaction, led to statistically significant shifts in microbial alpha diversity (Metrics: Richness and Shannon Diversity, all *P* > 0.05). Data represent a 2 × 3 factorial design with vaccination status (vaccinated vs unvaccinated) and iron supplementation (control, FeSO₄, SQM Iron). Boxplots show median, interquartile range, and individual data points (*n* = 5 birds per treatment group, one bird sampled per pen across five replicate pens per treatment).

Overall, vaccination and iron supplementation influenced cecal microbial communities in distinct ways. Alpha diversity, measured by observed richness and Shannon diversity, did not differ significantly across vaccination or supplementation treatments, nor was there evidence of an interaction effect (Fig. 3A and B; Tables 1 and 2; Scheirer–Ray–Hare test, all *P* > 0.05).

**TABLE 1:** Results from Scheirer-Ray-Hare test (non-parametric two-way ANOVA) examining the effects of vaccination status and iron supplementation on the microbial community richness (ASVs) of cecal microbiomes of female broilers^*a*^^,^^*b*^

Effect	df	Sum Sq	*H*	*P*-value
Vaccination	1	0.21	0.003	0.957
Iron supplementation	2	106.98	1.477	0.478
Vaccination × iron supplementation	2	245.42	3.388	0.184
Residual	23	1,675.60	–	–
Total	28	2,028.21	–	–

^*a*^ Shannon diversity in poultry cecal contents. The analysis included 29 samples across six treatment groups in a 2 × 3 factorial design. Effect = source of variation; df = degrees of freedom; Sum Sq = sum of squares; *H* = test statistic; *P*-value = probability value. No significant main effects or interactions were detected (all *P* > 0.05).

^*b*^ – indicates NA values, as *H* and *P*-values are not available for the residual and total effects.

**TABLE 2:** Scheirer-Ray-Hare test (non-parametric two-way ANOVA) examining the effects of vaccination status and iron supplementation on the Shannon diversity of cecal microbiomes of female broilers^*a*^^,^^*b*^

Effect	df	Sum Sq	*H*	*P*-value
Vaccination	1	88.86	1.226	0.268
Iron supplementation	2	19.51	0.269	0.874
Vaccination × iron supplementation	2	8.74	0.121	0.942
Residual	23	1,908.4	–	–
Total	28	2,025.51	–	–

^*a*^ The analysis included 29 samples across six treatment groups in a 2 × 3 factorial design. Effect = source of variation; df = degrees of freedom; Sum Sq = sum of squares; *H* = test statistic; *P*-value = probability value. No significant main effects or interactions were detected (all *P* > 0.05).

^*b*^ – indicates NA values, as *H* and *P*-values are not available for the residual and total effects.

### Beta diversity (Bray–Curtis, community composition)

Vaccination, iron supplementation, and their interaction significantly affected microbial community structure. For Bray–Curtis β-diversity, both vaccination and iron supplementation produced statistically significant main effects (PERMANOVA; vaccination: *F* = 2.65, *R*² = 0.07, *P* = 0.001; iron supplementation *F* = 3.43, *R*² = 0.18, *P* = 0.001), and significant interactive effects (*F* = 2.36, *R*² = 0.13, *P* = 0.001) (Table 3). *Post-hoc* pairwise PERMANOVA comparisons with Bonferroni adjustment for multiple comparisons identified a significant shift in microbial community composition between the vaccinated animals receiving standard feed (controls) and those supplemented with SQM Iron (*F* = 5.67, *p*_adj_ = 0.05), suggesting that SQM Iron supplementation exerts a measurable impact on the gut microbiota of vaccinated birds. In contrast, no other treatment comparisons were statistically significant after adjustment for multiple testing, underscoring the specificity of the SQM Iron effect.

**TABLE 3:** Permutational multivariate analysis of variance (PERMANOVA) testing the effects of vaccination and iron supplementation on the Beta diversity of cecal microbiomes of female broilers^*a*^^,^^*d*^

Effect	df	Sum Sq	*R*²^*c*^	*F*	*P*-value
Vaccination^*b*^	1	0.2328	0.071	2.65	0.001
Iron supplementation^*c*^	2	0.6032	0.184	3.43	0.001
Vaccination × iron supplementation	2	0.4143	0.127	2.36	0.001
Residual	23	2.0221	0.618	–	–
Total	28	3.2723	1.000	–	–

^*a*^ Using Bray-Curtis dissimilarity. Significant effects were set at *P* ≤ 0.05 with all main effects and interactions showing significant differences in community composition.

^*b*^ Birds were vaccinated with live-attenuated *Salmonella *vaccine (AviPro Megan Vac 1).

^*c*^ Diet was supplemented with iron polysaccharide complex (QualiTech, Inc., MN) and ferrous sulfate as the iron sources.

^*d*^ – indicates NA values, as *H* and *P*-values are not available for the residual and total effects.

**Fig 4:**
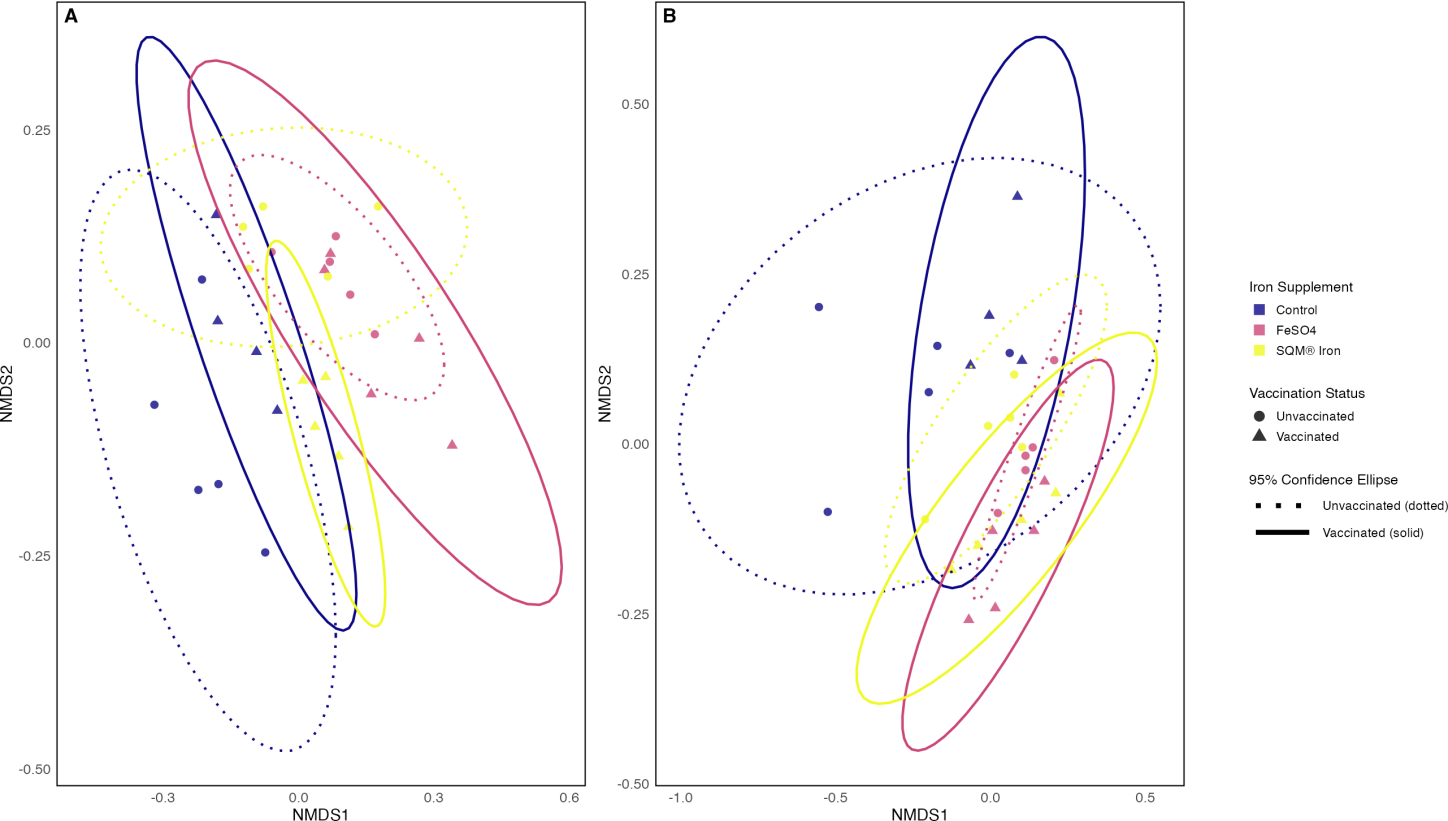
Both vaccination and iron supplementation and their interaction differentially shape microbial community structure. Beta diversity of gut microbiota across vaccination and iron supplementation treatments. NMDS ordination plots show microbial community dissimilarity patterns across treatment groups, with individual samples represented as points colored by iron supplementation treatment (Control, FeSO₄, SQM Iron) and shaped by vaccination status (circles = unvaccinated, triangles = vaccinated). Confidence ellipses (95%) indicate within-group variation for each treatment combination. (**A**) Pairwise Bray–Curtis dissimilarities were derived from ASV profiles standardized by rarefaction (4D NMDS solution, stress = 0.095). (**B**) Jaccard dissimilarity based on ASV presence/absence patterns (4D NMDS solution, stress = 0.081). For both panels, PERMANOVA was used to test for main effects of vaccination and iron supplementation and their interaction, followed by *post-hoc* pairwise comparisons with Bonferroni correction for multiple testing. Statistical results are reported in Tables 3 and 4 and in the main text.

NMDS ordination (Fig. 4A) provided a clear visualization of treatment-driven shifts in the microbial community. These findings indicate that the impact of iron supplementation on microbial community structure depended on vaccination status.

### Jaccard β-diversity (presence/absence)

Jaccard β-diversity analyses highlighted significant differences in the presence or absence of taxa in the cecal microbiome across treatments. For Jaccard β-diversity, both vaccination and iron supplementation produced statistically significant main effects (PERMANOVA; vaccination: *F* = 1.73, *R*² = 0.05, *P* = 0.002; iron supplementation *F* = 2.03, *R*² = 0.13, *P* = 0.001), as well as significant interactive effects (*F* = 1.71, *R*² = 0.106, *P* = 0.001) (Table 4). Jaccard β-diversity was calculated based on ASVs presence/absence data (after rarefaction) and visualized using NMDS (4D solution, stress = 0.081). Recall, unlike Bray–Curtis analyses (Fig. 4A), Jaccard β-diversity metrics reflect differences in the presence or absence of taxa rather than their relative abundance. These findings indicate that the impact of iron supplementation on the presence and absence of different taxa depended on vaccination status (Fig. 4B).

**TABLE 4:** Permutational multivariate analysis of variance (PERMANOVA) testing the effects of vaccination and iron supplementation on the presence or absence of specific microbes using Jaccard β-diversity (presence or absence)^*a*^^,^^*d*^

Effect	df	Sum Sq	*R*²^*c*^	*F*	*P*-value
Vaccination^*b*^	1	0.3414	0.054	1.73	0.002
Iron supplementation^*c*^	2	0.7985	0.126	2.03	0.001
Vaccination × iron supplementation	2	0.6749	0.106	1.71	0.001
Residual	23	4.5336	0.714	–	–
Total	28	6.3485	1.000	–	–

^*a*^ Significant effects were set at *P* ≤ 0.05 with all main effects and interactions showing significant differences in community composition.

^*b*^ Birds were vaccinated with live-attenuated *Salmonella *vaccine (AviPro Megan Vac 1).

^*c*^ Diet was supplemented with iron polysaccharide complex (QualiTech, MN, USA) and ferrous sulfate as the iron sources.

^*d*^ – indicates NA values, as *H* and *P*-values are not available for the residual and total effects.

### Differential abundance analysis

While Bacteroidota remained the dominant phylum across all treatment groups, several low-abundance taxa exhibited notable changes in response to vaccination, iron supplementation, and their interaction. Although relatively few of these changes were statistically significant (*q-*values > 0.05), likely due to limited sample sizes, the corresponding effect sizes suggest biologically meaningful shifts in microbial community structure.

**Fig 5:**
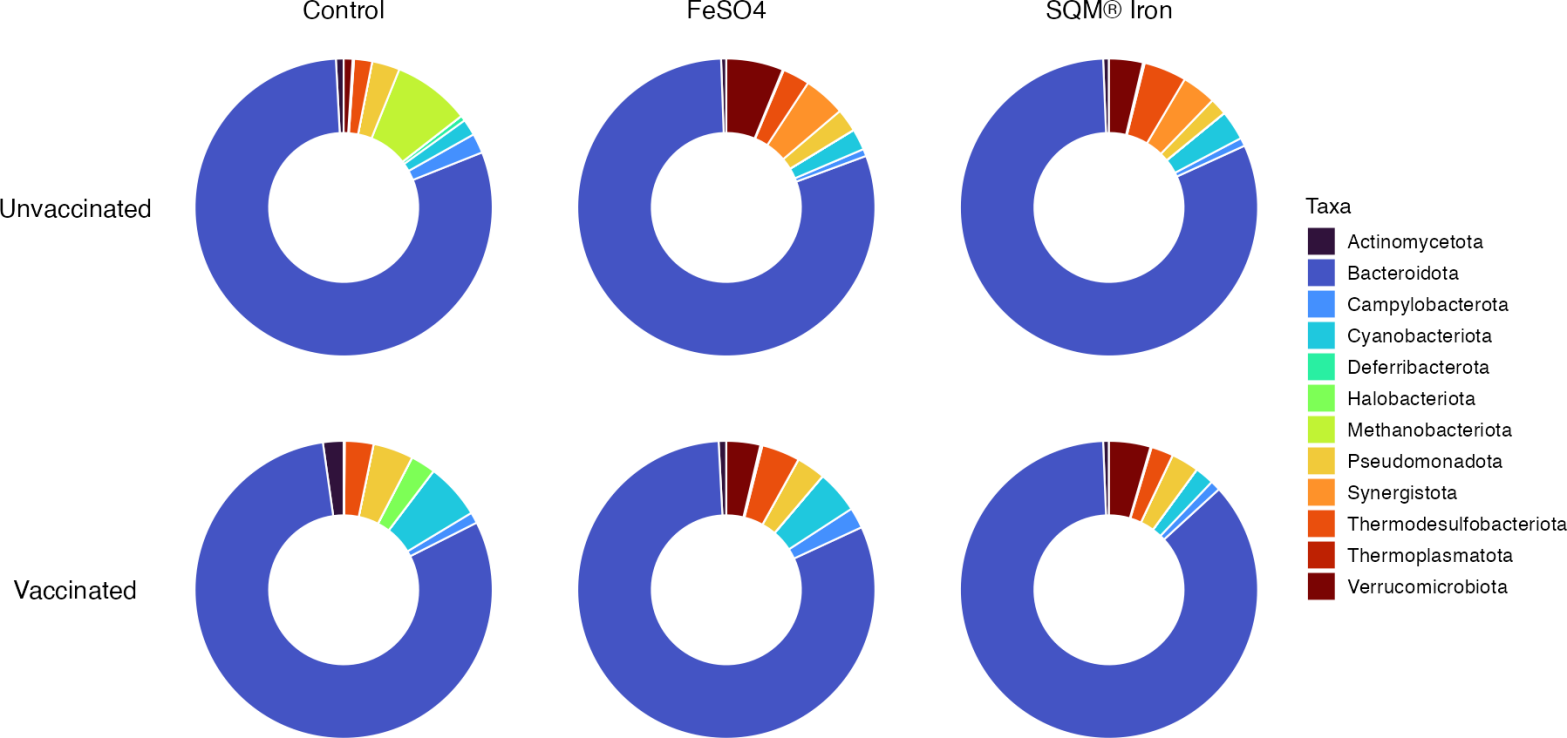
Relative abundance of bacterial phyla in the gut microbiota of poultry across vaccination and iron supplementation treatments. Donut plots display the mean relative abundance of classified bacterial taxa at the phylum level, stratified by vaccination status (rows) and iron supplementation treatment (columns). Data represent classified taxa only, with unclassified phyla excluded and the remaining phyla rescaled to 100% within each treatment group. Bacteroidota dominate the microbial community across all treatment groups, comprising the majority of the gut microbiota, while other phyla contribute smaller but variable proportions. Colors represent individual phyla as shown in the legend, with all detected and classified phyla included in the visualization.

At the phylum level, both vaccination and iron supplementation influenced the relative abundances of several minor microbial lineages (Fig. 5). None of these changes were statistically significant (ANCOM-BC2; *q-*values > 0.05). However, the relatively large effect sizes suggest that these low-abundance (rare) phyla may be responsive to both immunological and nutritional interventions. These changes were largely driven by *Cyanobacteriota*, *Campylobacterota*, *Thermodesulfobacteriota*, and *Verrucomicrobiota*. Vaccination alone had notable effects on several microbial taxa though, again, none reached statistical significance. At the phylum level, vaccination was associated with a large decrease in *Verrucomicrobiota* (–1.61, *q* > 0.05), with this phylum being approximately three times more abundant in unvaccinated birds. Two other phyla showed medium effect sizes: *Cyanobacteriota* (+0.86) was more abundant in vaccinated birds, while *Actinomycetota* (+0.53) also showed increased abundance with vaccination, though neither change was statistically significant (*q*-values > 0.05). (Table S2). The interaction between vaccination and SQM Iron drove additional shifts, including increases in *Verrucomicrobiota* (+1.44), alongside reductions in *Cyanobacteriota* (–1.39)*, Campylobacterota* (–1.00), and *Thermodesulfobacteriota* (–0.89) (Table S3).

**Fig 6:**
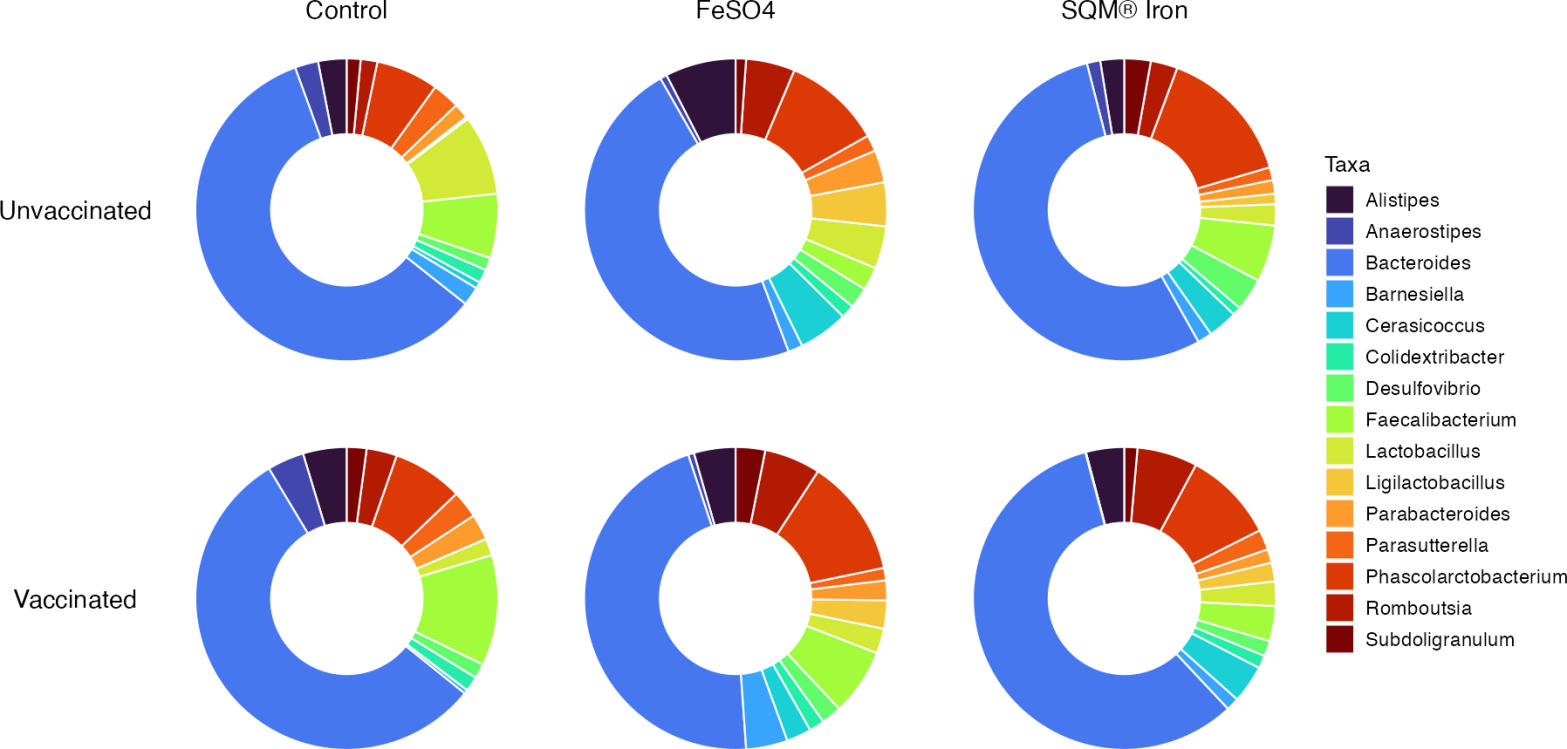
Relative abundance of dominant bacterial genera in the gut microbiota of poultry across vaccination and iron supplementation treatments. Donut plots display the mean relative abundance of the most prevalent classified bacterial taxa at the genus level, stratified by vaccination status (rows) and iron supplementation treatment (columns). Data represent the top 15 most abundant genera based on overall prevalence, with unclassified genera excluded and displayed genera rescaled to 100% within each treatment group. *Bacteroides* is the most abundant genus across all treatment groups, followed by other key genera including *Faecalibacterium*, *Phascolarctobacterium*, and *Lactobacillus*. Notable treatment-specific variations are observed in the relative abundances of minor genera. Colors represent individual genera as shown in the legend, with only the top 15 most prevalent genera shown.

At the genus level, vaccination, iron supplementation, and their interaction produced distinct shifts in microbial composition, with iron supplementation, particularly SQM Iron, exerting the most pronounced effects (Fig. 6). These changes were observed across both dominant and rare taxa, with several reaching statistical significance.

Vaccination alone tended toward minor enrichments in several genera within the Bacillota phylum, including *Merdibacter* (1.78), *Thomasclavelia* (1.66), *Ligilactobacillus* (1.03), *Pseudoflavonifractor* (1.51), and *Catenibacillus* (1.47). Although none of these changes were statistically significant (*q-*values > 0.05), the effect sizes were relatively large, suggesting biologically meaningful shifts (Table S4).

Iron supplementation alone also produced formulation-specific shifts in microbial composition. These effects were particularly evident among rare taxa, suggesting that iron availability influences not only dominant genera (Fig. 6) but also less abundant members of the microbiome with potential functional relevance (Table 5). *Pygmaiobacter* and *Odoribacter* were the only genera consistently enriched by both iron formulations. For *Pygmaiobacter*, the increase was stronger in SQM Iron-treated birds (+2.23) compared to FeSO₄ (+1.94), while *Odoribacter showed* a slightly higher response to FeSO₄ (+1.25) relative to SQM Iron (+1.15). In other words, the model predicts that *Pygmaiobacter* was ~4 times more abundant with SQM Iron relative to controls. Additionally, FeSO₄ supplementation significantly increased *Ligilactobacillus*, *Butyricimonas*, and *Odoribacter*, while significantly decreasing *Bacillus* (Table 5). SQM Iron significantly elevated *Catenibacillus*, *Odoribacter,* and *Desulfovibrio* and significantly reduced *Sutterella*, *Streptococcus*, *Anaerofilum*, and *Oscillospira* (Table 6).

**TABLE 5:** Genera *Ligilactobacillus*, *Butyricimonas*, and *Odoribacter* are significantly more abundant in FeSO₄-supplemented birds than controls, regardless of vaccination status^*a*^

Genus	Log2FC	SE	Effect size	*P*-value	*q*-value	Direction
*Ligilactobacillus*	2.456	0.386	Large	0.000005	0.000	Higher in FeSO_4_
*Pygmaiobacter*	1.936	–^*b*^	Large	1.000000	1.000	Higher in FeSO_4_
*Bacillus*	−1.628	0.214	Large	0.000628	0.012	Higher in control
*Butyricimonas*	1.334	0.344	Large	0.001338	0.019	Higher in FeSO_4_
*Odoribacter*	1.251	0.314	Large	0.000621	0.012	Higher in FeSO_4_

^*a*^ Results from a subset of the ANCOMBC2 analysis identifying genera with significant differences in abundance (*q* < 0.05) between iron sulfate (FeSO_4_) supplemented birds and control birds across vaccination groups. Taxon = genus name; lfc = log2 fold change (positive values indicate higher abundance in FeSO_4_ treatment, negative values indicate higher abundance in control); se = standard error of log2 fold change; *P*-value = uncorrected probability value; *q*-value = false discovery rate adjusted *P*-value; Effect Size = magnitude classification (small, medium, large based on |lfc| thresholds); Direction = interpretation of fold change direction. Analysis controlled for vaccination status and identified four genera with significant differential abundance: *Ligilactobacillus*, *Butyricimonas*, and *Odoribacter* were significantly more abundant in FeSO_4_-supplemented birds, while *Bacillus* was significantly more abundant in control birds. *Pygmalobacteria* were rare (or absent) and included here given the relatively larger increase in FeSO_4_ treatments.

^*b*^ –, not applicable.

**TABLE 6:** Variation in microbial genera between SQM Iron supplementation and control treatments^*a*^

Taxon	Log2FC	SE	Effect size	*P*-value	*q*-value	Direction
*Pygmaiobacter*	2.232	0.092	Large	0.026221	0.147	Higher in SQM Iron
*Catenibacillus*	1.203	0.142	Large	0.000062	0.002	Higher in SQM Iron
*Sutterella*	−1.178	0.112	Large	0.000002	0.000	Higher in control
*Odoribacter*	1.145	0.320	Large	0.001700	0.016	Higher in SQM Iron
*Streptococcus*	−1.143	0.293	Large	0.001300	0.014	Higher in control
*Desulfovibrio*	0.895	0.263	Medium	0.003000	0.024	Higher in SQM Iron
*Anaerofilum*	−0.680	0.105	Medium	0.000350	0.006	Higher in control
*Oscillospira*	−0.577	0.092	Medium	0.000760	0.011	Higher in control

^*a*^ Results from a subset of the full ANCOMBC2 analysis identifying genera with significant differences in abundance (*q* < 0.05) between SQM Iron supplemented birds and control birds while controlling for vaccination status. Taxon = genus name; Log2FC = log2 fold change (positive values indicate higher abundance in SQM Iron treatment, negative values indicate higher abundance in control); se = standard error of log2 fold change; *P*-value = uncorrected probability value; *q*-value = false discovery rate adjusted *P*-value; Effect Size = magnitude classification (small, medium, large based on |lfc| thresholds); Direction = interpretation of fold change direction. Analysis controlled for vaccination status and identified seven genera with significant differential abundance: *Catenibacillus*, *Odoribacter*, and *Desulfovibrio* were significantly more abundant in SQM Iron-supplemented birds, while *Sutterella*, *Streptococcus*, *Anaerofilum*, and *Oscillospira* were significantly more abundant in control birds.

The interaction between vaccination and iron supplementation revealed unique microbial shifts not observed under either treatment alone. No genus-level changes from the interaction reached statistical significance (*q-*values > 0.05), but again, the effect sizes suggest potentially biologically meaningful shifts in microbial function and composition. Several SCFA-producing genera, including *Anaerostipes* (–2.64), *Catenibacillus* (–1.75), and *Faecalibacterium* (–1.29), were markedly reduced in birds receiving both interventions, suggesting a potential suppressive interaction that may dampen fermentative and anti-inflammatory microbial functions. In contrast, the combined treatment enriched *Barnesiella* (+1.38), *Lactobacillus* (+1.48), *Staphylococcus* (+2.66), and *Streptococcus* (+1.20).

Together, these findings suggest that vaccination and iron supplementation may exert synergistic or additive effects on specific gut microbiota members, highlighting their potential for targeted microbial modulation strategies.

## DISCUSSION

Our results demonstrate that the interplay between vaccination and nutritional supplementation shapes the poultry cecal microbiome in ways that neither treatment achieves in isolation. Specifically, this interaction induces distinct taxonomic shifts across multiple scales, ranging from the phylum to the genus level. While *Bacteroidota* remained the dominant phylum across all treatment groups, the observed changes in low-abundance taxa suggest that these interventions could potentially exert biologically meaningful pressures on the microbial community structure (28, 29). This variation, though not statistically significant, likely reflects the early-stage ecological responses of the cecum to immunological and nutritional stimuli. The following sections explore how individual and interactive treatment effects drive these subtle yet potentially consequential taxonomic changes.

Vaccination as a standalone intervention induced a notable shift in the cecal environment, primarily characterized by the enrichment of taxa often associated with gut homeostasis (54, 55). Vaccination alone promoted shifts in several phyla, including *Campylobacterota* and *Thermodesulfobacteriota*, while *Verrucomicrobiota* was more abundant in unvaccinated birds. Most notably, vaccination was associated with an enrichment of several genera within the phylum *Bacillota*, a dominant group in the poultry gut that encompasses numerous short-chain fatty acid producers and immune-modulating taxa (56, 57). Genera such as *Merdibacter*, *Thomasclavelia*, *Ligilactobacillus*, *Pseudoflavonifractor*, and *Catenibacillus* were all markedly increased in vaccinated birds, suggesting these taxa may constitute a “core immunoresponsive microbiome” (56–58). Many of these genera are recognized for their roles in fermentation, nutrient metabolism, and mucosal barrier support (57–60). The consistent promotion of these beneficial *Bacillota* suggests that immune stimulation via vaccination may serve as a selective pressure that stabilizes the microbial community and reinforces the host’s functional intestinal defenses.

The specific formulation of iron supplementation serves as a primary driver of microbial community structure, with both forms eliciting distinct taxonomic perturbations. For instance, *Odoribacter*, *Butyricimonas*, and *Ligilactobacillus* were significantly enriched in birds receiving FeSO₄ supplementation, while *Catenibacillus*, *Desulfovibrio,* and *Odoribacter* were significantly elevated in SQM Iron-treated birds. In contrast, *Streptococcus,* a pathogen associated with opportunistic infections (61), showed higher abundance in controls compared to birds receiving SQM Iron (fold change = −1.14). These microbial dynamics could reflect a “relief of suppression” rather than direct iron support for rare genera. For instance, by masking iron availability, SQM Iron might prevent pathogens from activating iron-triggered virulence genes (such as those regulated by Fur) which, in turn, might limit the ability of pathogens like *E. coli* to outcompete other taxa (62, 63). Under these conditions, rare genera do not necessarily require specialized iron-acquisition mechanisms to thrive. They simply benefit from a more equitable competitive landscape where pathogen virulence is diminished by restricted access to free dietary iron (11, 56). Finely tuning the bioavailability of iron supplementation, therefore, represents an exciting opportunity to develop win-win solutions for poultry and other animals (7, 8, 21).

The combined effects of vaccination and iron supplementation revealed unique microbial shifts that were not observed under either treatment alone. Most notably, the treatment combination appeared to suppress several short-chain fatty acid (SCFA)-producing genera that are often considered beneficial, including *Anaerostipes* (–2.64 SQM Iron), *Catenibacillus* (–1.75 SQM Iron × Vaccine), and *Faecalibacterium* (–1.29 SQM Iron × Vaccine). Additionally, the iron-specific interactions with the vaccine revealed complex effects on other taxa, concurrently enriching generally beneficial genera such as *Barnesiella* (+1.38 SQM Iron × Vaccine, +2.55 FeSO₄ × Vacccine) and *Lactobacillus* (+1.48 SQM Iron × Vaccine), and some potentially less desirable taxa. Specifically, *Staphylococcus* showed a large interaction effect with SQM Iron (+2.66). *Streptococcus* also had a positive interaction (+1.20 SQM × Vaccine). While certain species within these latter genera are associated with opportunistic infections, the clinical significance of these shifts in broilers remains unclear; 16S rRNA gene sequencing lacks the resolution to distinguish pathogenic from commensal strains. Consequently, future studies employing an integrated approach using shotgun metagenomics, culture-based assays, and health outcome monitoring are necessary to determine if these taxonomic shifts carry implications for bird health (64). Ultimately, these results highlight the need to evaluate the synergistic or antagonistic outcomes of combined nutritional and immunological interventions (65–68) and underscore the complexity of treatment-host–microbiota interactions (25, 27).

While our study provides a broad, pen-level overview of microbial shifts, we recognize that assessing the variability in the microbiomes of individual birds represents an important next step in this research. Due to logistical constraints, we were only able to sample a single bird per pen, which limits our ability to examine fine-scale individual-level variation in the cecal microbiota. However, this approach provides a first step in assessing treatment-level signals and remains consistent with numerous studies in poultry research where the pen is the unit of measure (69–71). As seen in other studies, our alpha diversity results showed no significant differences, suggesting that the cecal environment is similar between birds (72). This finding indicates that while variation in pen conditions certainly is important, all else being equal, the effects of bird age (73–76), weight (77), and experimental treatments (78) typically far exceed pen-level variation. Therefore, our findings should be interpreted as treatment-level signals that reflect the combined impact of dietary iron and *Salmonella* vaccination on the cecal microbial community.

Our study characterized microbial dynamics in mature birds (day 49), 35 days after the administration of the vaccine booster. A critical next step involves longitudinal studies to track these microbial dynamics over time, particularly to determine if SQM Iron, or other forms of nutrient bioavailability, can act synergistically with vaccines to further limit colonization by pathogenic *Salmonella* serovars that are critical public health threats. At the conclusion of this current study, we tested for the presence of *Salmonella* isolates using a culture-based assay of the cecal contents. In agreement with the results of our 16S rRNA sequencing data, there was no evidence for the presence of *Salmonella* at this sampling location. These results are consistent with the known ecology of the avian gut; *Salmonella* colonization typically occurs early in the life cycle before the establishment of a mature microbiome that can provide competitive exclusion against pathogens (56, 79). Consequently, future research focusing on earlier developmental timepoints will be essential to elucidate whether SQM Iron functions through anti-infection, anti-growth, or anti-transmission mechanisms (66, 80).

The timing required to capture any interactive effects with vaccine colonization and persistence may be more challenging. While some studies indicate that vaccine strains could persist for extended periods, even post processing (81), the majority of studies suggest the persistence of *Salmonella* vaccine strains remains highly variable, with detectable persistence ranging from 2 days to 10 weeks (82–85). This variability likely reflects differences in vaccination strains, dosing schedules, sampling strategies, and the specific biological objectives of each vaccine. Indeed, prolonged colonization of the intestinal tract is not the primary objective for the AviPro MeganVac 1 vaccine; rather, its efficacy is tied to the systemic translocation of this attenuated strain to immune-responsive tissues like the liver and spleen.

Because translocation requires the activation of certain virulence genes, we conducted a parallel study to determine if restricted iron bioavailability from SQM Iron might inhibit this early process. In other words, could the reduced bioavailability of iron in the gut inhibit virulence gene expression in the vaccine strain and thus translocation to immune-responsive tissues? Our results on day 5 post-vaccination confirmed that vaccine strain prevalence exceeded 80% in both SQM Iron and FeSO₄ treatment groups, with no significant interaction observed between iron source and vaccination status (Jendza, unpublished data). These data indicate that, while the formulation of dietary iron can cause taxonomic shifts in the microbiome, at least in certain rare taxa, it does not interfere with the initial colonization or systemic immunogenic potential of the vaccine. We propose that longitudinal studies monitoring both microbiome dynamics and vaccine strain recovery could provide vital insights into the mechanisms modulating vaccine efficacy.

In the meantime, the results presented here underscore the potential of integrated management strategies, a direction increasingly advocated for by the poultry industry and policymakers (5, 68, 86, 87). Collectively, these findings reinforce the concept that aligning immunological interventions with targeted micronutrient supplementation could unlock new opportunities for optimizing gut health. As global demand for poultry continues to rise, such integrated strategies will be essential for providing “win-win” solutions that improve bird health, welfare, and performance while simultaneously enhancing food safety.

## Data Availability

All data and analysis code have been deposited in GitHub (https://github.com/jhite-eco-epi/SQM-Iron-Avipro-Megan-Vac-1-Vaccine-Poultry-Microbiome). The 16S rRNA raw sequence reads generated in this study have been deposited in the NCBI Sequence Read Archive (SRA) database and are publicly available under accession number PRJNA1299482.

## SUPPLEMENTAL MATERIAL

Supplemental material aem.00127-26-s0001.docx Fig. S1 and S2; Tables S1 to S4.

ASM does not own the copyrights to Supplemental Material that may be linked to, or accessed through, an article. The authors have granted ASM a non-exclusive, world-wide license to publish the Supplemental Material files. Please contact the corresponding author directly for reuse.
