# Supplementary material for: Diet–vaccine interactions: SQM Iron and Salmonella vaccination shape poultry gut microbiota
Source: Appl Environ Microbiol. 2026 Apr 14;92(5):e00127-26. doi: 10.1128/aem.00127-26 (PMC13188920; doi:10.1128/aem.00127-26)
Supplement: Supplemental material — Fig. S1 and S2; Tables S1 to S4. [file aem.00127-26-s0001.docx]

**Supplementary Materials**

**Diet–Vaccine Interactions: SQM® Iron and *Salmonella* Vaccination Shape Poultry Gut Microbiota**

Eldon O. Ager^1^†, Colette A. Nickodem^2^†, Jessica Brown^3^, Joshua Jendza^4^, Eric Neeno-Eckwall^2^, Marisa Schuldes^2^, Dana K. Dittoe^5^, and Jessica L. Hite^1^*

^1^Department of Integrative Biology, University of Wisconsin-Madison

^2^Department of Pathobiological Sciences, University of Wisconsin-Madison

^3^Department of Animal and Dairy Sciences, University of Wisconsin-Madison

^4^QualiTech, LLC., MN

^5^Department of Animal and Dairy Sciences, University of Wisconsin-Madison

†These authors contributed equally to this work.

*Correspondence: jhite2@wisc.edu

**
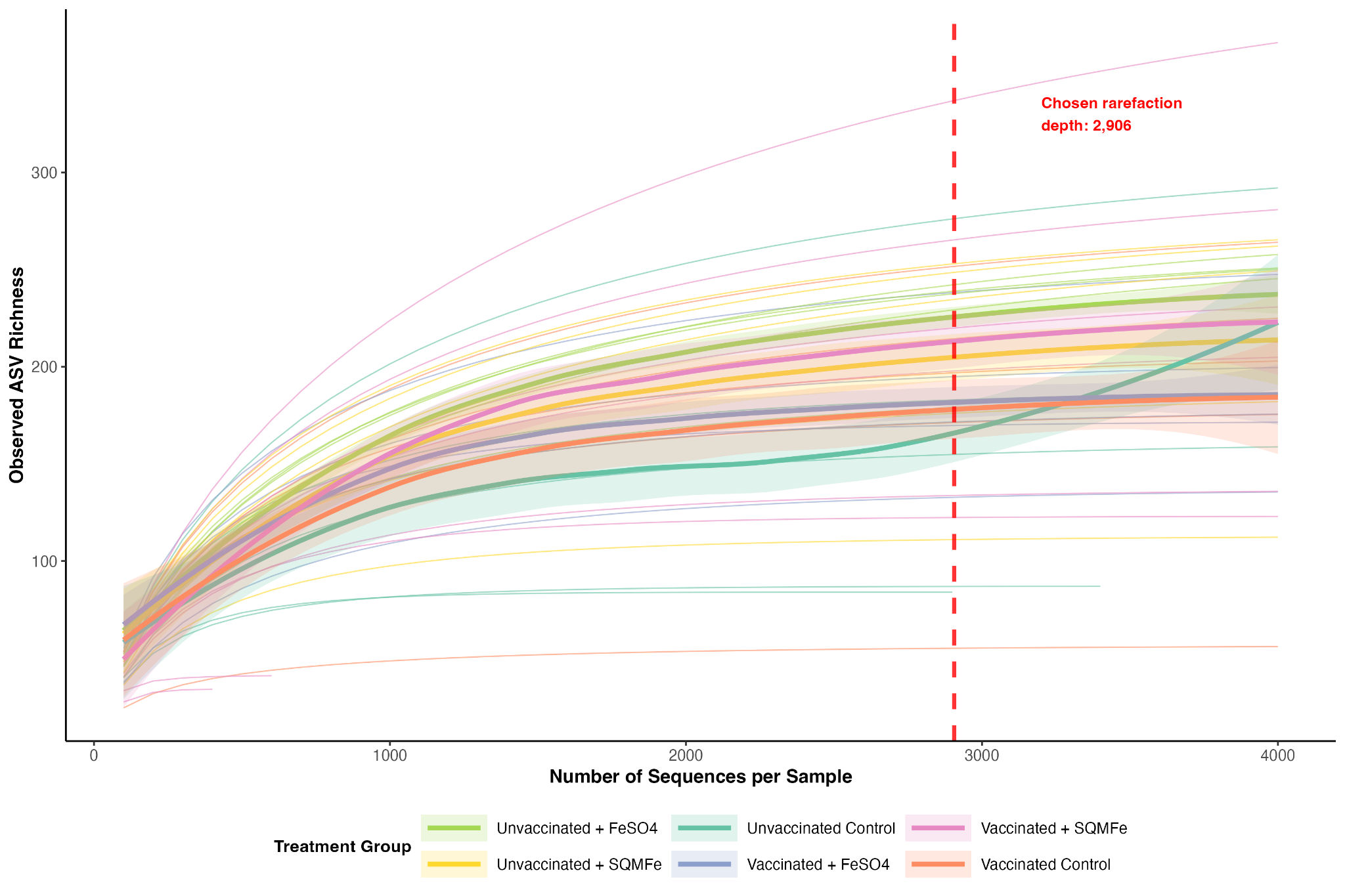
**

Supplementary Figure S1. Rarefaction curves of all samples. Number of observed ASVs plotted against sequencing depth (number of reads). Each line represents one sample (n = 30).


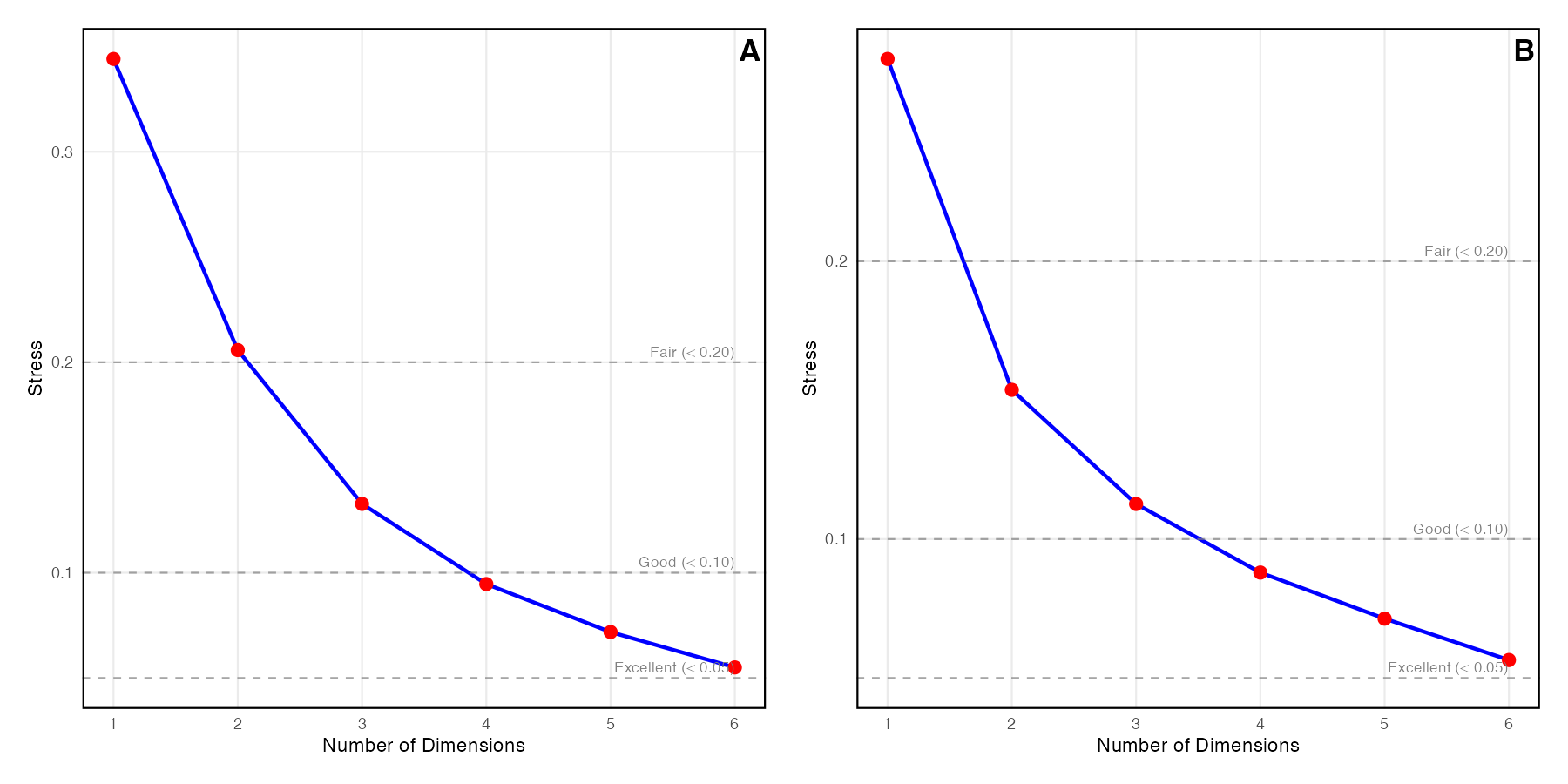


Supplementary Figure S2: NMDS stress analysis for beta diversity dimensionality optimization. Stress values plotted against number of dimensions for (A) Bray-Curtis dissimilarity based on ASV abundance data and (B) Jaccard dissimilarity based on ASV presence/absence data. Both analyses used rarefaction data (2,906 sequences per sample) from 29 samples across six treatment groups. Horizontal dashed lines indicate stress quality thresholds: excellent (< 0.05), good (< 0.10), and fair (< 0.20). Blue lines show stress reduction with increasing dimensions, with red points marking specific dimensional solutions. Both distance metrics support 4-dimensional NMDS solutions with good stress values (Bray-Curtis: 0.095; Jaccard: 0.088), providing adequate representation of beta diversity patterns for visualization while maintaining interpretability. The 4D solutions were selected for ordination plots based on the balance between model fit (stress < 0.10) and dimensional parsimony.

Supplementary Table S1. Composition and nutritional values of experimental diets for Ross 708 female broilers supplemented with different iron sources (g/kg). The study used a three-phase feeding program spanning 49 days, with nutritionally equivalent diets formulated for starter (0-14 days), grower (14-28 days), and finisher (28-49 days) phases.

|  | Starter (0-14 d) | | Grower (14-28 d) | | Finisher (28-49 d) | |
| --- | --- | --- | --- | --- | --- | --- |
| Ingredients | SQM® Iron¹ | FeSO₄² | SQM® Iron | FeSO₄ | SQM® Iron | FeSO₄ |
| Corn | 663.7 | 663.7 | 671.13 | 671.13 | 658.59 | 658.59 |
| Soybean meal (47.9% CP) | 237.26 | 237.26 | 183.61 | 183.61 | 151.28 | 151.28 |
| DDGS³ (27% CP) | 40 | 40 | 80 | 80 | 120 | 120 |
| Meat & bone meal (46% CP) | 40 | 40 | 40 | 40 | 40 | 40 |
| Soybean oil | - | - | 4.72 | 4.72 | 13.41 | 13.41 |
| Limestone | 4.66 | 4.66 | 4.4 | 4.4 | 4.12 | 4.12 |
| Dicalcium phosphate | 4.59 | 4.59 | 2.96 | 2.96 | 1.16 | 1.16 |
| Salt | 4.27 | 4.27 | 4.09 | 4.09 | 3.91 | 3.91 |
| Choline chloride (60%) | 1.03 | 1.03 | 0.94 | 0.94 | 0.76 | 0.76 |
| Vitamin premix⁴ | 0.5 | 0.5 | 0.5 | 0.5 | 0.5 | 0.5 |
| SQM® Iron premix⁵ | 1.26 | - | 1.26 | - | 1.26 | - |
| FeSO₄ premix⁶ | - | 1.26 | - | 1.26 | - | 1.26 |
| L-Lysine HCl | 0.85 | 0.85 | 3.33 | 3.33 | 2.95 | 2.95 |
| DL-Methionine (99%) | 1.68 | 1.68 | 2.24 | 2.24 | 1.57 | 1.57 |
| L-Threonine (98.5%) | - | - | 0.64 | 0.64 | 0.31 | 0.31 |
| Phytase⁷ | 0.18 | 0.18 | 0.18 | 0.18 | 0.18 | 0.18 |
| Total | 1000 | 1000 | 1000 | 1000 | 1000 | 1000 |
|  |  |  |  |  |  |  |
| Analyzed iron content (mg/kg) | 143.88 | 143.88 | 118.77 | 118.77 | 93.06 | 93.06 |
|  |  |  |  |  |  |  |
| Calculated nutrients |  |  |  |  |  |  |
| Crude protein (%) | 19.5 | 19.5 | 18.4 | 18.4 | 17.76 | 17.76 |
| ME poultry (kcal/kg) | 3027 | 3027 | 3080 | 3080 | 3135 | 3135 |
| Calcium (%) | 0.85 | 0.85 | 0.8 | 0.8 | 0.75 | 0.75 |
| Available phosphorus (%) | 0.42 | 0.42 | 0.4 | 0.4 | 0.38 | 0.38 |
| Sodium (%) | 0.22 | 0.22 | 0.22 | 0.22 | 0.22 | 0.22 |
| SID⁸ Lysine (%) | 0.93 | 0.93 | 1 | 1 | 0.9 | 0.9 |
| SID⁸ Methionine (%) | 0.47 | 0.47 | 0.51 | 0.51 | 0.43 | 0.43 |
| SID⁸ Met+Cys (%) | 0.73 | 0.73 | 0.75 | 0.75 | 0.68 | 0.68 |
| SID⁸ Threonine (%) | 0.65 | 0.65 | 0.65 | 0.65 | 0.59 | 0.59 |
| SID⁸ Valine (%) | 0.85 | 0.85 | 0.78 | 0.78 | 0.74 | 0.74 |

¹SQM® Iron = Polysaccharide iron complex (SQM® Iron, QualiTech). ²FeSO₄ = Ferrous sulfate. ^3^DDGS = Dried Distillers Grains with Solubles.^4^The vitamin premix provided the following quantities of vitamins per kilogram of diet: vitamin A, 4,248-4,744 IU; vitamin D₃, 1,500 IU; vitamin E, 10.7-12.2 IU; vitamin B₁₂, 7.8 μg; biotin, 0.04-0.08 mg; menadione, 0.75 mg; thiamine, 0.66-0.78 mg; riboflavin, 3.0-3.3 mg; pantothenic acid, 5.4-5.9 mg; pyridoxine, 1.1-1.4 mg; niacin, 22.2-24.6 mg; folic acid, 0.33-0.36 mg. ^5^The SQM® Iron trace mineral premix contained (per kg of diet): zinc (from zinc sulfate), 120 mg; manganese (from manganese sulfate), 120 mg; iron (from SQM® Iron), 60 mg; copper (from copper sulfate), 16 mg; iodine (from calcium iodide), 1.25 mg; selenium, 0.3 mg. ^6^The ferrous sulfate trace mineral premix contained (per kg of diet): zinc (from zinc sulfate), 120 mg; manganese (from manganese sulfate), 120 mg; iron (from ferrous sulfate), 60 mg; copper (from copper sulfate), 16 mg; iodine (from calcium iodide), 1.25 mg; selenium, 0.3 mg. ^7^Quantum Blue Phytase. ^8^SID = Standardized ileal digestible.

Supplementary Table S2: Phylum-level differential abundance analysis for vaccination effects. Results from ANCOM-BC2 analysis examining phylum-level abundance differences between vaccinated and unvaccinated groups with significance threshold set at *q* < 0.05. Taxon = phylum name; Log2FC = log2 fold change (positive values indicate higher abundance in vaccinated animals, negative values indicate higher abundance in unvaccinated animals); SE = standard error of the log2 fold change estimate; p-value = raw p-value from statistical test; q-value = false discovery rate adjusted p-value; Effect Size = magnitude classification (Small, Medium, Large based on |Log2FC| thresholds); Direction = interpretation of fold change direction relative to vaccination status. Analysis identified eight phyla with varying responses to vaccination, with effect sizes ranging from small to large, but none reaching statistical significance after multiple testing correction. Verrucomicrobiota showed the largest vaccination effect (Log2FC = -1.613, Large effect) with higher abundance in unvaccinated animals, while Cyanobacteriota and Actinomycetota demonstrated medium-sized positive responses to vaccination. The remaining five phyla (Thermoplasmatota, Bacteroidota, Thermodesulfobacteriota, Campylobacterota, and Pseudomonadota) showed small effect sizes with mixed directional responses, suggesting subtle phylum-level microbiome shifts associated with vaccination status

| Taxon | Log2FC | SE | p-value | q-value | Effect Size | Direction |
| --- | --- | --- | --- | --- | --- | --- |
| Verrucomicrobiota | -1.613 | 0.692 | 0.031000 | 0.248 | Large | Higher in Unvaccinated |
| Cyanobacteriota | 0.858 | 0.635 | 0.190000 | 0.758 | Medium | Higher in Vaccinated |
| Actinomycetota | 0.531 | 0.795 | 0.511000 | 0.882 | Medium | Higher in Vaccinated |
| Thermoplasmatota | -0.308 | 0.608 | 0.621000 | 0.882 | Small | Higher in Unvaccinated |
| Bacteroidota | -0.302 | 0.612 | 0.626000 | 0.882 | Small | Higher in Unvaccinated |
| Thermodesulfobacteriota | -0.161 | 0.642 | 0.804000 | 0.882 | Small | Higher in Unvaccinated |
| Campylobacterota | 0.131 | 0.873 | 0.882000 | 0.882 | Small | Higher in Vaccinated |
| Pseudomonadota | 0.108 | 0.631 | 0.865000 | 0.882 | Small | Higher in Vaccinated |

Supplementary Table S3: Phylum-level vaccination × SQM® iron interaction effects. Results from ANCOM-BC2 analysis examining interaction effects between vaccination status and SQM® iron supplementation at the phylum level with significance threshold set at *q* < 0.05. Taxon = phylum name; Log2FC = log2 fold change for the interaction term (positive values indicate vaccination effects are stronger in SQM® iron group, negative values indicate vaccination effects are weaker in SQM® iron group compared to control); SE = standard error of the interaction effect estimate; p-value = raw p-value from statistical test; q-value = false discovery rate adjusted p-value; Effect Size = magnitude classification (Small, Medium, Large based on |Log2FC| thresholds); Direction = interpretation of how vaccination effects differ between SQM® iron and control groups. Analysis identified eight phyla with varying interaction patterns, with effect sizes ranging from small to large, but none reaching statistical significance after correction for multiple testing. Verrucomicrobiota showed the largest positive interaction effect (Log2FC = 1.438, Large effect), indicating vaccination effects are substantially stronger in the SQM® iron treatment group. Conversely, Cyanobacteriota and Campylobacterota demonstrated large negative interaction effects (Log2FC = -1.391 and -1.004, respectively).

| Taxon | Log2FC | SE | p-value | q-value | Effect Size | Direction |
| --- | --- | --- | --- | --- | --- | --- |
| Verrucomicrobiota | 1.438 | 1.194 | 0.243247 | 0.809 | Large | Vaccination effect stronger in SQM® |
| Cyanobacteriota | -1.391 | 0.913 | 0.141085 | 0.809 | Large | Vaccination effect weaker in SQM® |
| Campylobacterota | -1.004 | 1.179 | 0.404306 | 0.809 | Large | Vaccination effect weaker in SQM® |
| Thermodesulfobacteriota | -0.893 | 0.984 | 0.373398 | 0.809 | Medium | Vaccination effect weaker in SQM® |
| Actinomycetota | -0.599 | 1.164 | 0.611672 | 0.923 | Medium | Vaccination effect weaker in SQM® |
| Thermoplasmatota | 0.391 | 0.967 | 0.692591 | 0.923 | Small | Vaccination effect stronger in SQM® |
| Pseudomonadota | 0.197 | 0.881 | 0.825247 | 0.943 | Small | Vaccination effect stronger in SQM® |
| Bacteroidota | -0.065 | 0.938 | 0.945241 | 0.945 | Small | Vaccination effect weaker in SQM® |

Supplementary Table S4: Genus-level differential abundance analysis for vaccination effects. Results from ANCOM-BC2 analysis examining genus-level abundance differences between vaccinated and unvaccinated groups with significance threshold set at q < 0.05. Taxon = genus name; Log2FC = log2 fold change (positive values indicate higher abundance in vaccinated animals, negative values indicate higher abundance in unvaccinated birds); SE = standard error of the log2 fold change estimate; p-value = p-value from statistical test; q-value = false discovery rate adjusted p-value; Effect Size = magnitude classification (Small, Medium, Large based on |Log2FC| thresholds); Direction = interpretation of fold change direction relative to vaccination status. Analysis identified six genera with large effect sizes showing consistently higher abundance in vaccinated animals, though none reached statistical significance after multiple testing correction. *Merdibacter* showed the largest vaccination effect (Log2FC = 1.784), followed by *Thomasclavelia* (Log2FC = 1.659) and *Mailhella* (Log2FC = 1.537).

| Taxon | Log2FC | SE | p-value | q-value | Effect Size | Direction |
| --- | --- | --- | --- | --- | --- | --- |
| *Merdibacter* | 1.784 | 0.491 | 0.036000 | 0.287 | Large | Higher in Vaccinated |
| *Thomasclavelia* | 1.659 | 0.589 | 0.010000 | 0.240 | Large | Higher in Vaccinated |
| *Mailhella* | 1.537 | 0.523 | 0.009000 | 0.240 | Large | Higher in Vaccinated |
| *Pseudoflavonifractor* | 1.506 | 0.533 |  | 1.000 | Large | Higher in Vaccinated |
| *Catenibacillus* | 1.472 | 0.491 | 0.020000 | 0.240 | Large | Higher in Vaccinated |
| *Ligilactobacillus* | 1.027 | 0.384 | 0.015000 | 0.240 | Large | Higher in Vaccinated |
